# Effectiveness of Online Self-Help for Suicidal Thoughts: Results of a Randomised Controlled Trial

**DOI:** 10.1371/journal.pone.0090118

**Published:** 2014-02-27

**Authors:** Bregje A. J. van Spijker, Annemieke van Straten, Ad J. F. M. Kerkhof

**Affiliations:** 1 Centre for Mental Health Research, Australian National University, Canberra, Australia; 2 Department of Clinical Psychology and the EMGO Institute for Health and Care Research, Faculty of Psychology and Education, VU University Amsterdam, Amsterdam, the Netherlands; Iran University of Medical Sciences, Iran (Republic of Islamic)

## Abstract

**Background:**

Many people with suicidal thoughts do not receive treatment. The Internet can be used to reach more people in need of support.

**Objective:**

To test the effectiveness of unguided online self-help to reduce suicidal thoughts.

**Method:**

236 adults with mild to moderate suicidal thoughts were randomised to the intervention (n = 116) or a waitlist control group (n = 120). Assessments took place at baseline, and 2, 4 and 6 weeks later. Primary outcome was suicidal thoughts. Secondary outcomes were depressive symptoms, anxiety, hopelessness, worry, and health status.

**Results:**

The intervention group showed a small significant effect in reducing suicidal thoughts (d = 0.28). Effects were more pronounced for those with a history of repeated suicide attempts. There was also a significant reduction in worry (d = 0.33). All other secondary outcomes showed small but non-significant improvements.

**Conclusions:**

Although effect sizes were small, the reach of the internet could enable this intervention to help many people reduce their suicidal thoughts.

**Trial Registration:**

Netherlands Trial Register NTR1689

## Introduction

Although effective treatments exist [1], [2], [3], [4], [5], [6] 44% of suicidal people in high income countries do not receive treatment [7]. Barriers to seeking help include a preference for self-reliance, believing in spontaneous recovery, thinking the problem is not severe, believing that treatment will not be effective, fear of stigma, shame, and prejudice against, and/or negative experiences with, healthcare providers [7], [8]. Providing anonymous help online may address some of these obstacles [9], [10]. Moreover, people who receive mental health treatment could benefit from an additional online intervention [11].

The majority of people with suicidal thoughts meet criteria for a psychiatric disorder, most commonly depression and to a lesser extent anxiety [12], [13]. In addition, hopelessness has frequently been associated with suicidality [14], [15]. More recently, associations between worry and rumination and suicidal thoughts have been described. Specifically the repetitive character of suicidal thoughts shows similarities with worry and rumination [16], [17].

Web-based interventions have been found effective for a range of mental disorders, e.g. depression [18], [19], anxiety [18], [20], and problem drinking [21]. Most of these web-based interventions are based on Cognitive Behavioural Therapy (CBT), which is relatively easy to adapt to an (online) self-help format due to its structured approach. Evidence that online treatment can reduce suicidal ideation is now also emerging [22], [23]. This paper describes a randomised controlled trial (RCT) comparing unguided web-based self-help for suicidal thoughts with a waitlist control group. It was hypothesised that the intervention would be better in reducing suicidal thoughts expecting a small effect size given previous findings that effect sizes for unguided self-help are generally modest and lower than those for guided self-help [19]. In addition, improvements were expected on all secondary outcomes (depression, hopelessness, worry, anxiety, and health status).

## Methods

The protocol for this trial and supporting CONSORT checklist are available as supporting information; see Protocol S1 and Checklist S1.

### Design, Setting, and Participants

Participants were recruited between October 2009 and November 2010 through websites (e.g. www.113Online.nl), newspapers and Google Adwords advertising. Participants needed to be aged at least 18, have access to the internet and e-mail, know Dutch well, have mild to moderate suicidal thoughts, and not be severely depressed. Mild to moderate suicidal thoughts were defined as a score between 1 and 26 on the Beck Scale for Suicide Ideation (BSS) [24]. Severe depression was defined as a score >39 on the Beck Depression Inventory (BDI) [25]. These two criteria were established in consultation with clinical experts, and were employed because people with severe symptoms are likely to need more intensive care and may not be focused enough to work through the intervention. These criteria were also used for the safety procedure (see below). Already receiving help, regardless of the source, was not an exclusion criterion.

Eligibility was assessed using an online application procedure. Respondents who exceeded cut-off scores (i.e. BSS>26 and/or BDI>39) were referred to other (mental health) services using an automated response. Eligible respondents were requested to fill in their e-mail address, gender, age, educational level, living situation (alone or together), current use of mental health care, and the importance they placed on remaining anonymous during the study. Subsequently, they were sent an information brochure, consent form, and a link to the baseline questionnaire. Participants were required to disclose their identity and that of their general practitioner (GP) in order to be able to apply the safety procedure.

Participants were randomised to the intervention or the waitlist control condition by an independent researcher using a block design (20 per block) and stratified by gender. The randomisation outcome was communicated by e-mail with either a log-in code for the intervention or a link to a website with general information on suicidality for the control group. Six weeks after randomisation, the control group received log-in codes for the intervention website. The study protocol is described in more detail elsewhere [26] and was registered in the Netherlands Trial Register, NTR1689 (http://www.trialregister.nl/trialreg/admin/rctview.asp?TC=1689).

### Ethics Statement

This study was approved by the Medical Ethics Committee of the VU University Medical Centre (registration number 2008/204). Written informed consent was obtained after the procedures had been fully explained.

### Safety of Participants

As this study involved participants at risk of suicide, a safety protocol was followed. Whenever participants in either condition exceeded cut-off scores of 26 on the BSS and/or 39 on the BDI, they were phoned, a risk assessment was done, and if necessary, their GP was contacted. These phone calls were made by a psychologist under supervision of a licensed clinical psychologist experienced in suicide prevention. Participants’ GPs were also contacted if a participant could not be reached [26].

### Intervention

This trial’s unguided self-help intervention is based on Cognitive Behavioural Therapy (CBT) [27], but also makes use of components of Dialectical Behavioural Therapy (DBT) [28], [29], Problem Solving Therapy (PST) [30], and Mindfulness Based Cognitive Therapy (MBCT) [31], [32]. All these treatment programs have evidence for their effectiveness in reducing suicidality [1], [2], [3], [6], [33].

The main goal of this intervention is helping participants decrease the frequency and intensity of their suicidal thoughts. Content was developed with the help of an expert team consisting of clinical psychologists and psychiatrists experienced in the treatment of suicidal people. A focus on controlled thinking, rather than thought cessation, should lead to reduced suicidal thinking. It consists of six modules, focusing on 1) the repetitive character of suicidal thoughts [16], 2) regulating intense emotions, 3) identifying automatic thoughts, 4) thinking patterns, 5) thought challenging, and 6) relapse prevention. Each module contains a theory section, a weekly assignment, a few ‘core exercises’ and several ‘optional exercises’. For example, the first module explains that suicidal thoughts can develop out of self-protection, as keeping on living may seem worse than dying. Similarities between worry and suicidal thinking are also outlined. The weekly assignment involves tallying suicide-related thoughts to obtain an idea of how often these occur, while the core exercises aim at learning to manage these repetitions better by introducing worry postponement. The optional exercises contain other strategies for managing suicidal thoughts, such as positive worrying, attentive breathing and seeking distraction. Participants follow one module per week and can receive up to six motivating automated e-mails. There is a FAQ function on the website via which questions can be asked. Participants are encouraged to complete one module per week and ideally spend 30 minutes per day on the program. A paper version of the intervention was given to five patients attending an outpatient mental health treatment facility in Amsterdam to obtain feedback, after which final improvements were made and the website was developed.

Participants in the control condition received access to a website constructed for this study providing information on suicidality such as prevalence rates and risk factors, taking about 15 minutes to read. In addition, common treatment options were listed and links to mental health care institutions were provided.

### Outcome Measures

Questionnaires were self-report and administered online. The primary outcome was the reduction of suicidal thoughts, assessed with the 21-item BSS [24] at baseline (T0), 2 and 4 weeks into the intervention (T1 and T2), and at post-test (6 weeks after baseline: T3). Total score of the BSS ranges from 0 to 38, with higher scores indicating more severe suicidal thoughts. The BSS has high internal reliability and moderate test-retest reliability [24].

Depressive symptoms, a secondary outcome, was also assessed at T0, T1, T2, and T3, using the BDI-II [34]. A total score between 0 and 13 indicates minimal depression, 14–19 mild depression, 20 to 28 moderate depression, and 29–63 severe depression. The BDI has good internal consistency [34].

Other secondary outcome measures were assessed only at T0 and T3. These included hopelessness (Beck Hopelessness Scale (BHS) [35], [36], score range 0–20), anxiety (Hospital Anxiety and Depression Scale (HADS-A) [37], score range 0–21), and worry (Penn State Worry Questionnaire Past Week (PSWQ-PW) [38], score range 0–90). A higher score on these questionnaires indicates more severe symptoms. Health status was measured using the thermometer of the EuroQol [39], ranging from 0 (worst imaginable health status) to 100 (best imaginable health status).

Finally, it must be noted that a follow-up measure took place 12 weeks after post-test (see also the study protocol [26]; see also Protocol S1), which will be described in a separate paper, and that cost-effectiveness outcomes have been published elsewhere [40], see also File S1.

### Power Analyses

Sample size was based on the expected effect on the primary outcome measure, i.e. the reduction of suicidal thoughts. In order to be able to detect an effect size of 0.35 with α = 0.05 and β = 0.80, 100 subjects were needed in each condition. Including an expected drop-out attrition rate of 20–30% in each group, the sample size was determined at 260.

### Statistical Analyses

Demographic and clinical characteristics of participants and people who declined participation were compared using t-tests and χ^2^ tests. The same procedures were used to test whether the control and intervention groups differed significantly on these characteristics at baseline.

Difference in drop out attrition rate [41] (i.e. drop out from the study) between both conditions was analysed using χ^2^ test. Subsequently, t-tests and χ^2^ tests were conducted to detect differences in baseline characteristics between participants who dropped out and those who did not. Next, multiple imputation was used to replace missing values.

Non-usage attrition [41] (i.e. drop out from the intervention) was analysed using χ^2^ tests and One-way Analysis of Variance (ANOVA) to detect differences at baseline between three groups based on adherence (within the intervention condition): 1) participants who did not start the intervention, 2) participants who completed one or two modules, and 3) participants who completed at least three modules.

Use of the safety procedure was evaluated for the entire study period by simple counts of the number of calls to participants and GPs. The number of participants that attempted suicide was based on self-reported attempts in the questionnaires. Differences in number of participants who attempted suicide between the intervention and the control group were tested using χ^2^ tests. No definition of attempted suicide was provided to participants, which means that the reported figures reflect participants’ own definition of what constitutes a suicide attempt.

For all outcome measures, mean change from baseline to post-test was analysed using t-tests on the multiply-imputed intention to treat (ITT) sample. Assumptions for parametric testing were checked and were found to be sufficient. Degrees of freedom were adjusted according to Barnard and Rubin [42]. Between-group effect sizes were calculated according to Cohen’s d. Also, a Bonferroni correction was applied to the analysis of the secondary outcome measures to control the overall Type 1 error rate. As 5 tests were done on secondary outcome measures, the α-level was adjusted to 0.01.

Subsequently, the primary outcome measure was analysed more thoroughly using a linear mixed model (LMM) to model change over time in suicidal thoughts, assuming a linear effect. The LMM procedure includes incomplete cases in the analysis and estimates their missing values by means of restricted maximum likelihood estimates. Time was included as a continuous variable, condition was treated as a fixed effect, and the intercept was included as a random effect. The LMM procedure assumes that data are missing at random (MAR) and was performed using a dataset without imputed values.

Finally, as it has previously been shown that response to an intervention may differ according to history of suicide attempt [43], an exploratory post hoc analysis was conducted on the primary outcome measure to examine treatment effects separately for those with no previous attempt at baseline (N = 137), those with one previous attempt at baseline (N = 39), and those with more than one previous attempt at baseline (N = 56) (using the imputed ITT dataset).

All analyses were conducted using IBM SPSS Statistics for windows version 20.0.

### Deviations from Study Protocol

In the protocol that was submitted to the ethics committee, severe suicidal thoughts were defined as a score ≥16 on the BSS and severe depression was defined at as score ≥29 on the BDI. Soon after the start of the trial, these criteria proved too stringent as the majority of respondents was excluded during screening. An amendment was submitted to the ethics committee proposing to change these to >26 for the BSS and >39 for the BDI. This was approved within one month after start of recruitment. Furthermore, the full length EuroQol was initially included [26] to have the potential to conduct cost-utility analyses, but it was decided that cost-effectiveness analyses were more appropriate, mainly because the EuroQol is designed to measure rather large improvements in health which are hard to achieve with a 6-week psychological self-help intervention (as this would not likely impact on the mobility, self-care, and pain questions of the EuroQol). In other words, our advancing insights led us to conclude that the full length EuroQol would not be sensitive to change in our trial and therefore focused on the cost-effectiveness analyses. Please note that we do report on health status as measured by the thermometer item of the EuroQol (see ‘outcome measures’). In addition, the Loneliness Scale included in the ethics protocol was not included in the final set of questionnaires in the interest of brevity, and insomnia was taken into account as a symptom of depression in the BDI and therefore not analysed separately. Finally, the Work and Social Adjustment scale was not analysed due to a clerical error in the admission of the scale which led to one of the items being omitted from the scale, invalidating its use.

## Results

### Participants

The enrolled sample size was smaller than anticipated (N = 236), but since the drop out attrition rate was smaller as well (with a maximum of 10.6% at T2), this did not affect the power.

**Figure 1:**
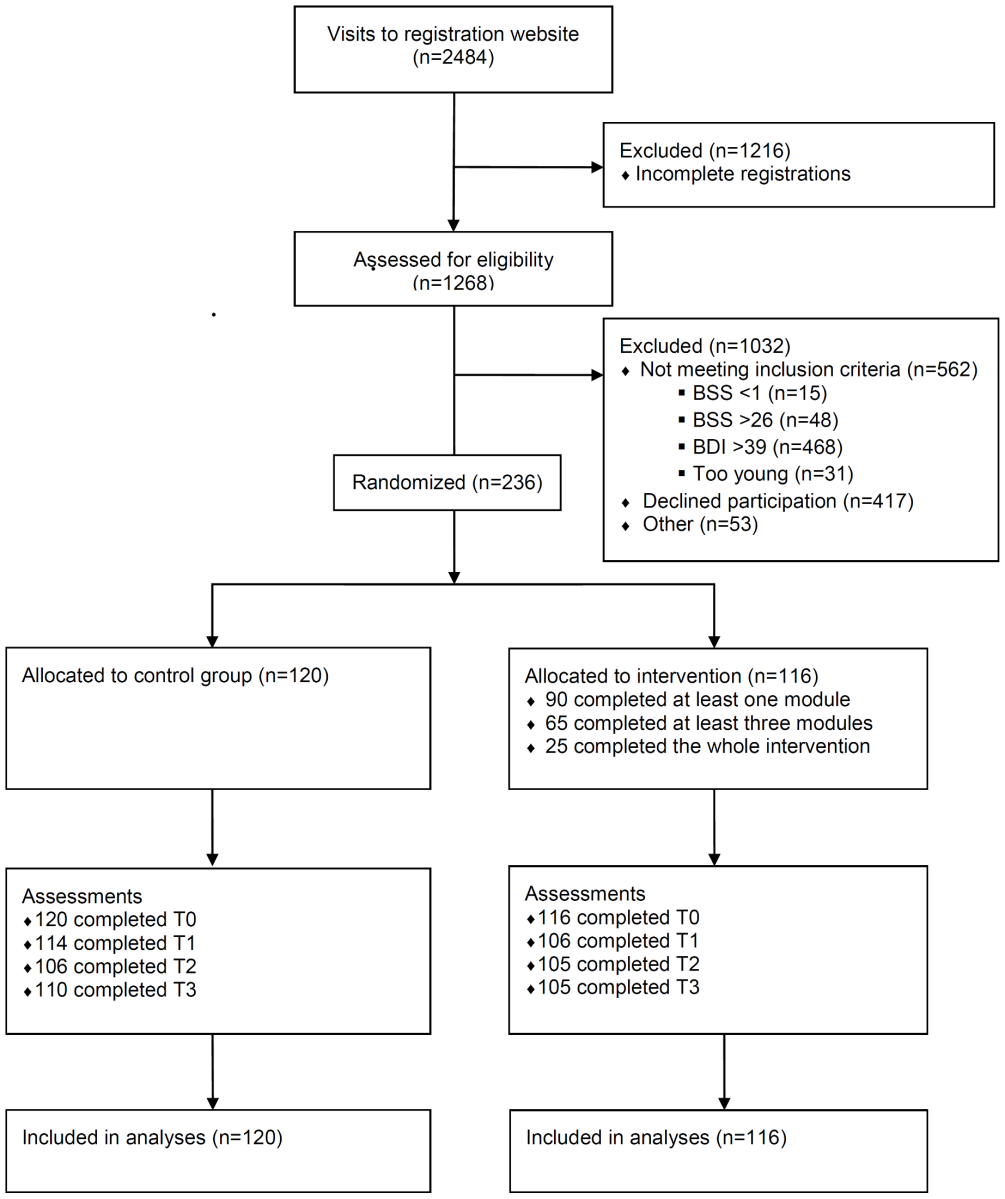
Participant flow through trial.

Figure 1 shows the participant flow through the trial. The registration website received 2,484 visits during the inclusion period. About half of these visits represented respondents who failed to complete the screening questionnaire (N = 1,216, 45.7%). The remaining 1,268 visitors completed the screening, after which almost half proved to be ineligible (N = 562, 44.3%), mainly due to severe depressive symptoms (N = 468, 83.3%). Another substantial proportion was eligible, but failed to return the informed consent form (N = 417, 32.9%). A small percentage of eligible respondents was excluded for various other reasons at this point, the most common being a failure to provide an email address, making it impossible to send the information brochure and consent form (N = 53, 4.2%). The remaining 236 eligible respondents returned their consent forms and were randomised.

An analysis comparing respondents who gave informed consent and thus became participants of the trial (N = 236) with those who declined (N = 417) indicated that study participants were slightly older (40.89 vs. 37.17 years, *t*(622) = 3.25, *p* = 0.001) and higher educated (χ^2^(3) = 7.81, *p* = 0.050). Moreover, participants rated anonymity less important than people who declined participation (39.8% and 61.9% respectively, (χ^2^(1) = 30.44, *p* = 0.000) and more often reported receiving some form of care (37.2% and 27.9% respectively for seeing a psychologist or psychiatrist, and 19.5% and 15.6% respectively for receiving another form of help, χ^2^(2) = 10.37, *p* = 0.006). There was no difference in severity of suicidal thoughts or depressive symptoms between participants and declined respondents.

Table 1 displays baseline characteristics for all participants randomised. The majority was female (N = 156; 66.1%), born in the Netherlands (N = 218; 94.0%) and had received education on an intermediate level (N = 112; 47.5%). A minority lived with a partner (N = 95; 40.3%) and had one or more children (N = 87; 37.5%). Mean age of the total sample was 40.93 (SD = 13.71) and half was in paid employment (N = 116; 50.0%). Less than half of the sample indicated not receiving any form of care at baseline (N = 100; 43.3%), while 37.3% was seeing a psychologist or a psychiatrist (N = 86) and 19.5% received some other form of care (N = 45). Regarding clinical characteristics, the average scores of the total sample at baseline reveal substantial levels of suicidal thoughts (M = 14.85, SD = 7.08), depression (M = 27.06, SD = 9.17), hopelessness (M = 14.38, SD = 3.73), anxiety (M = 10.37, SD = 3.70), worry (M = 57.80, SD = 11.27), and health status (M = 61.32, SD = 18.01). There were no differences at baseline between the two conditions regarding demographic or clinical characteristics.

**Table 1:** Baseline characteristics of total sample.

		Condition		
	Total (N = 236)	Control (N = 120)	Intervention (N = 116)	*p*
**Demographic characteristics**				
Female gender (N, %)	156 (66.1)	80 (66.7)	76 (65.5)	0.852
Age (M, SD)	40.93 (13.71)	41.39 (13.39)	40.46 (14.07)	0.602
Education (N, %)				
Lower	19 (8.1)	8 (6.7)	11 (9.5)	
Intermediate	112 (47.5)	52 (43.3)	60 (51.7)	0.365
Higher	90 (38.1)	51 (42.5)	39 (33.6)	
Other	15 (6.4)	9 (7.5)	6 (5.2)	
Living with a partner (N, %)	95 (40.3)	54 (45.0)	41 (35.3)	0.131
Has children (N, %)^1^	87 (37.5)	50 (42.0)	37 (32.7)	0.145
Born in the Netherlands (N, %)^1^	218 (94.0)	111 (93.3)	107 (94.7)	0.651
Paid employment (N, %)^1^	116 (50.0)	59 (49.6)	57 (50.4)	0.895
Receiving care (N, %)^2^				
No	100 (43.3)	53 (44.9)	47 (41.6)	
Yes, from psychologist or psychiatrist	86 (37.2)	41 (34.7)	45 (39.8)	0.727
Yes, other	45 (19.5)	24 (20.3)	21 (18.6)	
**Clinical characteristics**				
Suicidal thoughts (M, SD)	14.85 (7.08)	14.50 (7.33)	15.20 (6.82)	0.444
Attempted suicide (N, %)^1^				
Never	137 (59.1)	73 (61.3)	64 (56.7)	
Once	39 (16.8)	20 (16.8)	19 (16.8)	0.688
More than once	56 (24.1)	26 (21.8)	30 (26.5)	
Depressive symptoms (M, SD)	27.06 (9.17)	26.53 (9.04)	27.61 (9.31)	0.364
Hopelessness (M, SD)^1^	14.38 (3.73)	14.08 (3.90)	14.70 (3.53)	0.204
Worry (M, SD)^1^	57.80 (11.27)	56.87 (11.53)	58.78 (10.97)	0.199
Anxiety (M, SD)^1^	10.37 (3.70)	10.14 (3.86)	10.60 (3.52)	0.346
Health status (M, SD)^1^	61.32 (18.01)	62.55 (18.20)	60.04 (17.79)	0.289

1 Missing: N = 4, of which 1 in control and 3 in intervention group.

2 Missing: N = 5, of which 2 in control and 3 in intervention group.

### Attrition

Dropout attrition rates for the full sample were 6.8% (N = 16) at T1, 10.6% (N = 25) at T2, and 8.9% (N = 21) at T3. In total, 21 persons dropped out of the study, equally spread over the control (N = 10) and intervention (N = 11) groups (χ^2^(1) = 0.096, *p* = 0.757). Another eleven had intermittent missing values at T1, T2, or both, and were not included in the drop out analysis.

Reasons for dropout attrition included lack of time (N = 4), recovery of symptoms (N = 3), admission to a psychiatric hospital (N = 2), not finding the intervention useful (N = 3), participation in another study (N = 1), or were not specified (N = 8). Comparison of baseline characteristics showed that participants who dropped out felt more hopeless at baseline (M = 16.95 vs. M = 14.23; *t*(230) = 1.98, *p* = 0.049). For the remaining characteristics, no significant differences were found.

Analysing non-usage attrition in the intervention condition showed that 26 participants (22.4%) did not start the intervention. Twenty-five participants (21.6%) completed one or two modules and 65 (56.0%) completed at least three modules. ANOVA revealed no statistical differences in baseline characteristics between these groups. In addition, there were no differences in care received at baseline between these groups (χ^2^(4) = .014, *p* = 0.53). Participants in the intervention group reported having spent 10.5 hours on the whole intervention, i.e. an average of 15 minutes per day over the six weeks.

### Safety Procedure

During the study, participants who exceeded the cut-offs on the BSS and/or the BDI were called. Participants in the control condition were called more often than participants in the intervention condition (N = 31 vs. N = 19). In a number of cases, the GP was called because of high risk (N = 9 in the control group and N = 3 in the intervention group).

Based on self-report, eleven participants attempted suicide, of which seven were in the control group and four were in the intervention group (χ^2^(1) = 0.87, *p* = 0.351). No completed suicides occurred during the study.

### Main Outcome Measures

In table 2, the mean change between baseline and post-test in all continuous outcome measures for the intervention group, compared with the control group, are displayed. The results show significantly greater improvement in suicidal thoughts in the intervention group compared with the control group (from 15.2 to 10.7 versus 14.5 to 12.2: t(98) = −2.12, *p* = 0.036). The corresponding between group effect size for suicidal thoughts was 0.28.

**Table 2:** Mean change from baseline to post-test and effect sizes.

	Control (N = 120) (M, SD)	Intervention (N = 116) (M, SD)	d (95% CI)	p
Suicidal thoughts	2.30 (6.57)	4.47 (8.72)	**0.28 (0.03; 0.54)**	**0.036**
Depressive symptoms	1.82 (8.76)	3.93 (10.12)	0.22 (−0.03; 0.48)	0.086
Hopelessness	0.68 (3.61)	1.91 (4.95)	0.29 (0.03; 0.54)	0.029
Worry	2.12 (10.08)	5.48 (10.12)	**0.33 (0.08; 0.59)**	**0.010**
Anxiety	0.51 (3.29)	1.03 (3.88)	0.14 (−0.11; 0.40)	0.270
Health status*	3.00 (18.29)	−1.96 (19.71)	−0.26 (−0.52; 0.00)	0.045

•For health status, an increase in score (resulting in a negative difference between baseline and post-test) represents an improvement.

In addition, improvements were detected in all secondary outcome measures. For worry, a significant difference was found between the intervention group and the control group (t(186) = −2.60, p = 0.010). The corresponding between group effect size was 0.33. Although the tests for the other secondary outcome measures did not reach significance after applying a Bonferroni correction, they were in the expected direction. Between-group effect sizes for these secondary outcomes ranged from 0.14 for anxiety to 0.29 for hopelessness.

The results for suicidal thoughts were confirmed by the LMM procedure. Besides a significant improvement over time in both groups (F(1, 656) = 63.34, *p*<0.001), there was a significant time-by-group interaction effect (F(1, 656) = 8.38, *p* = 0.004), indicating a greater reduction of suicidal thoughts in the intervention group. Specifically, the average reduction in the intervention group (1.58 points on the BSS) was twice as much per time unit (i.e. two weeks) as in the control group (0.74 points on the BSS) (t(656) = 2.90, *p* = 0.004).

The post hoc exploratory subgroup analysis examining treatment effects according to history of attempted suicide revealed a significantly greater improvement in the intervention group (M = 5.43, SD = 9.09) compared to the control group (M = 0.43, SD = 8.67) in those who had attempted suicide more than once (t(174) = −2.02, *p* = 0.045; d = 0.56, 95% CI: 0.03–1.10). No such differences were found for those who had never attempted suicide or for those who had attempted only once.

## Discussion

This RCT found that web-based self-help can be effective in reducing suicidal thoughts. This effect seems more pronounced in participants with a history of repeated suicide attempts. The groups could no longer be compared at follow-up (12 weeks after post-test) because the control group obtained log in codes for the intervention at post-test, but improvements in the intervention group were generally maintained at follow-up (this will be described in a separate paper).

The results accord with what may be expected based on previous results for face-to-face cognitive therapy for suicidality [2], [3], and for unguided web-based treatment for related disorders [19]. Although unguided programs generally demonstrate lower effect sizes than guided ones [19], the unguided nature of this intervention was integral to the study. The rationale for this decision was based on facilitating implementation and dissemination processes in organizations and countries with limited finances. However, when means are available, guidance could be provided.

Results for the secondary outcome measures were also consistent with the hypotheses. Although a significant difference was only detected for worry, the intervention group generally showed greater improvement on these outcomes compared with the control group. The reduction in worry is consistent with the intervention’s focus on repetitive thinking in relation to suicidal thoughts. The finding that depressive symptoms are reduced by the intervention (although the effect was not significant) is consistent with recent research by Christensen et al. [22], who found that an online intervention for depression can reduce suicidal ideation. Although it is a common assumption that treating depression impacts on suicidality, there is currently not sufficient support for this in face-to-face settings [44]. More research is therefore needed to determine the potential mediating effects of improvements in depression on suicidality.

A design incorporating a safety procedure in which participants at risk were contacted and GPs were involved was an ethical condition for this trial. Also, the exclusion of severe suicidality and severe depression were part of the safety precautions. In hindsight, it is noteworthy that the majority of people who were excluded had severe depressive symptoms, but less severe suicidal thoughts. In future trials, it might be worth considering broader inclusion of this group, as they would appear to be a motivated subset of the population in need of help. Relatively few severe suicidal respondents were excluded, indicating that the study sample represents a fair share of the suicidal population. It is also notable that the safety procedures seemed to have had an effect on attrition, which was lower than anticipated, as participants who did not complete a questionnaire were contacted by phone as part of these procedures.

A limitation of this study is that many eligible respondents declined participation (32.9%), for which lack of anonymity is likely to have been important. It may thus be argued that the sample differed from the target population. Exclusion of people with severe depressive symptoms and severe suicidality also limits generalizability of the results. Furthermore, the fact that people who dropped out felt more hopeless at baseline may have influenced results. A third limitation is that the recruitment method was specific to the trial. In real world settings, recruitment for the intervention would depend on the context in which the intervention if offered. If, for example, this would be in the context of an online suicide prevention service, recruitment may mainly occus online and through (internal) referral, whereas if it would be offered within a secondary care mental health institution, clinicians would likely play a greater role in recruitment. Another limitation is that the trial was relatively small and randomisation may therefore not have dealt with all confounders. A fifth limitation concerns the finding that respondents with a history of multiple attempts benefitted more. This finding was based on a post hoc exploratory analysis rather than pre-defined analysis, and future trials should therefore be adequately powered and stratified according to history of attempts. A final limitation is that no formal psychiatric diagnoses were available. This approach is in line with the implementation goal of the intervention: that it should become available to people with suicidal thoughts in general, irrespective of the presence of a diagnosed disorder.

The outcomes of this project have a number of implications for the wider community. Firstly, these findings demonstrate that online self-help is a valid way of reaching people who might not otherwise seek help. Secondly, the fact that almost 57% of participants were receiving additional help implies that the intervention may work as an adjunct to regular care. Thirdly, there may potentially be a flow-on effect to the prevention of attempted and completed suicide, but this study cannot draw conclusions about the impact on suicidal behaviour. It is clinically relevant, and meaningful to sufferers, to reduce the psychological distress associated with suicidal thinking. Especially for those who are reluctant to access regular mental health services, it is better to have some form of help or support than none.

Since completion of the trial, the intervention has been implemented in www.113Online.nl [45], a Dutch suicide prevention platform that provides a range of services. 113Online currently provides support via email by trained volunteers or professionals alongside the self-help intervention if desired. Embedding the intervention in an integrated online suicide prevention platform also has the advantage that people can be referred to a more intensive or immediate form of support when needed.

Evidently, this trial’s results need to be confirmed in future studies. Web-based help can reach people who do not seek or receive adequate care due to attitudinal barriers or due to geopolitical barriers when living in a region where mental health care is less available or accessible. In addition, it can be useful as an adjunct to face to face treatment. Although this study’s effect size was small, the worldwide reach of the web and the low costs of unguided web-based self-help could enable it to help many people reduce their suicidal thoughts.

## Supporting Information

Protocol S1 **Trial protocol.**
(PDF)

Checklist S1 **CONSORT checklist.**
(DOCX)

File S1 **Publication with results cost-effectiveness analysis.**
(PDF)
